# Alignment of multiple metabolomics LC-MS datasets from disparate diseases to reveal fever-associated metabolites

**DOI:** 10.1371/journal.pntd.0011133

**Published:** 2023-07-24

**Authors:** Ana-Maria Năstase, Michael P. Barrett, Washington B. Cárdenas, Fernanda Bertuccez Cordeiro, Mildred Zambrano, Joyce Andrade, Juan Chang, Mary Regato, Eugenia Carrillo, Laura Botana, Javier Moreno, Clément Regnault, Kathryn Milne, Philip J. Spence, J. Alexandra Rowe, Simon Rogers

**Affiliations:** 1 School of Infection & Immunity, University of Glasgow, Glasgow, Scotland, United Kingdom; 2 Laboratorio para Investigaciones Biomedicas, Facultad de Ciencias de la Vida, Escuela Superior Politécnica del Litoral (ESPOL), Guayaquil, Ecuador; 3 Servicio de Infectología e Epidemiología, Hospital de Niños Dr. Roberto Gilbert, Guayaquil, Ecuador; 4 Instituto Nacional de Investigación en Salud Pública (INSPI), Guayaquil, Ecuador; 5 WHO Collaborating Centre for Leishmaniasis, National Centre for Microbiology, Instituto de Salud Carlos III, Madrid, Spain; 6 Institute of Immunology and Infection Research, University of Edinburgh, Edinburgh, United Kingdom; 7 School of Computing Science, University of Glasgow, Glasgow, Scotland, United Kingdom; KU Leuven, BELGIUM

## Abstract

Acute febrile illnesses are still a major cause of mortality and morbidity globally, particularly in low to middle income countries. The aim of this study was to determine any possible metabolic commonalities of patients infected with disparate pathogens that cause fever. Three liquid chromatography-mass spectrometry (LC-MS) datasets investigating the metabolic effects of malaria, leishmaniasis and Zika virus infection were used. The retention time (RT) drift between the datasets was determined using landmarks obtained from the internal standards generally used in the quality control of the LC-MS experiments. Fitted Gaussian Process models (GPs) were used to perform a high level correction of the RT drift between the experiments, which was followed by standard peakset alignment between the samples with corrected RTs of the three LC-MS datasets. Statistical analysis, annotation and pathway analysis of the integrated peaksets were subsequently performed. Metabolic dysregulation patterns common across the datasets were identified, with kynurenine pathway being the most affected pathway between all three fever-associated datasets.

## Author summary

Fever-associated infectious diseases are still a major cause of concern in low to middle income countries. Inappropriate treatment of misdiagnosed diseases can contribute to the selection of drug resistant microbes. Therefore, improved diagnostics of febrile patients and specific biomarker discovery to support new diagnostics is desirable. Metabolomics studies can provide the necessary information which leads to the discovery of biomarkers. In this study we have investigated three different metabolomics datasets; including those for malaria, leishmaniasis and Zika virus infection, all associated with fever. We aimed to integrate these metabolomics datasets to determine metabolites which behave in the same way in different infectious diseases. One of the challenges in integrating metabolomics datasets is a non-linear drift which occurs between them in terms of retention time. In this case, we proposed to correct this drift by using a supervised machine learning algorithm called Gaussian Process Regression. Following the integration or alignment of the datasets statistical analysis and annotation of the metabolites was performed. We identified several metabolites which acted in a similar manner across the datasets, specifically those found in the kynurenine pathway of tryptophan metabolism.

## Introduction

Many infectious diseases are characterised by fever: a generic host response to numerous microbial pathogens. Fever is associated with the hypothalamus which, in response to the activation of cyclooxygenase-2 (COX-2), releases prostaglandin E2 (PGE2), triggering a systemic increase in body temperature which can have microbicidal effects [1]. Although fever has a protective effect, acute febrile illnesses are still a major cause of mortality and morbidity globally, particularly in low to middle income countries [2]. The failure to correctly diagnose a specific disease associated with fever is partly responsible for this. Inappropriate treatment of misdiagnosed diseases can contribute to the selection of drug resistant microbes. For example, in many parts of Africa, fever is assumed to be due to malaria and treated with anti-malarial drugs. In cases where the patient may actually not have been infected with malaria parasites, but subsequently became infected while the drug concentration was waning, a selective pressure on resistant mutants could have been imposed [3]. Therefore, improved diagnostics of febrile patients and specific biomarker discovery to support new diagnostics is desirable.

Recently, increasing numbers of studies on fever-associated diseases using untargeted metabolomics with mass spectrometry coupled to high performance liquid chromatography (LC-MS) have emerged [4–8]. LC-MS is a sensitive approach to identifying metabolite markers and for providing a comprehensive coverage of the metabolome, as it enables the separation and measurement of thousands of discrete chemical compounds. Following the LC-MS peak detection process, a list of ions characterised by the chromatographic retention time (RT), mass to charge ratio (m/z) and intensity is obtained.

When performed on individual disease states, however, distinguishing between metabolites associated generically with fever, and others specific for particular diseases becomes a challenge. Thus, this study aimed to search for common perturbations to compounds across a set of fever associated diseases simultaneously, in order to identify metabolites generically associated with fever or disease severity. In order to achieve this, a method for integration of multiple LC-MS datasets through peakset alignment was developed.

The alignment process, also referred to as correspondence, for which algorithms are categorized into either direct matching or warping algorithms, has been extensively studied, mainly in the context of multiple injections within the same experiment [9]. This is due to the fact that although the LC-MS instrumentation and methodology are robust and well established, measurement variability can still appear, resulting in non-linear shifts especially in RT. There are two types of variability sources which can appear in an LC-MS experiment and cause mis-alignment and/or cross-alignment [10]: system variation and component level variation [9]. The system variation is usually consistent throughout the whole run and may be caused by factors such as the apparatus itself, the column, system stability and temperature [11]. Component level variation, by contrast, is specific to a single analyte or a group of analytes, so it cannot be modelled using monotonic functions. In order to reduce the RT variability within an experiment, a set of known metabolites, or internal standards, is run at various points during an LC-MS experiment as part of the quality control process [11, 12]. Using internal standards enables the drift in compound intensity and especially RT to be tracked throughout every LC-MS run. Therefore, the internal standards metabolites can be used as landmarks for retention time correction as the m/z for each compound detected using LC-MS is constant.

In this study, the information provided by the internal standards runs was used to determine and correct the RT drift between injections of different LC-MS experiments by modelling it using Gaussian Process (GP) regression, a supervised machine learning approach [13]. GP regressions are a non-parametric approach to modelling data and they differ from standard regression models in that they do not require any assumptions about a particular parametric form for the function being modelled. Fitted GPs were used to perform high level correction of retention times between experiments, after which standard alignment was performed.

The algorithm was developed here specifically to determine whether any metabolites could be identified that changed in abundance in similar ways across a series of distinct fever-associated diseases. These include Zika virus infection in patients from Ecuador [14], Leishmaniasis patients from Spain [15] and uncomplicated malaria infected volunteers from the UK [16]. The samples had been run previously using the same LC-MS platform (Glasgow Polyomics, University of Glasgow, UK). By seeking metabolites for which the variation in abundance followed common trends in different datasets, we aimed to determine disease-generic metabolites that could assist in both understanding the pathophysiology of infectious disease, and also in identifying metabolites that may be fever rather than specific disease related.

## Materials and methods

### Datasets

Three LC-MS datasets –D_*Z*_, D_*M*_, D_*VL*_– analysed at Glasgow Polyomics metabolomics facility (University of Glasgow, UK) were used for the cross-experimental integration in this study. Detailed information regarding the sample collection process for each dataset is included in the Supporting Information (S1 File). All three experiments were designed for detecting the differences between the serum metabolic profiles of healthy controls and infected patients diagnosed by gold-standard methods. In total, there were 74 samples (37 controls and 37 disease samples). Detailed information about each LC-MS experiment is presented in Table 1.

**Table 1:** LC-MS datasets details.

	D_*Z*_	D_*M*_	D_*VL*_
Infectious Disease	Zika virus [14]	Malaria [16]	Visceral Leishmaniasis [15]
Study Type	Case-control	Intervention	Case-control
LC Column/MS Platform	pHILIC/Q-Exactive	pHILIC/Q-Exactive	pHILIC/Q-Exactive
LC-MS Run Length (min)	26	26	46
Date Analysed	2018	2016	2018
Healthy Controls	10	7	20
Infected Patients	10	7	20
Internal Standards Sets	3	3	3
MS2 data	Yes	Yes	Yes

Information about each LC-MS experiment, including the disease studied, type of study, number of controls and patients, LC-MS platform used, date when the samples from the LC-MS experiment were run and availability of fragmentation (MS2) data, are presented.

#### LC-MS platform

The experiments were run at different time points (Table 1) using the same LC-MS platform: Thermo Orbitrap QExactive (Thermo Fisher Scientific) mass spectrometer coupled with a Dionex UltiMate 3000 RSLC system (Thermo Fisher Scientific, Hemel Hempstead, UK) using a ZIC-pHILIC column (150 mm × 4.6 mm, 5 *μ*m column, Merck SeQuant). While the same flow rate was used for all three datasets, the length of the run differed for *D*_*VL*_, which lasted longer than the other two datasets. Data from both positive (+ve) and negative (−ve) electrospray ionisation (ESI) mode was obtained.

#### Tandem mass spectrometry data

Fragmentation of the pooled samples within each experiment was performed using higher energy C-trap dissociation (HCD) at a normalised collision energy (NCE) of 60. These were analysed with TopN data-dependent acquisition (DDA) fragmentation strategies, resulting in tandem mass spectrometry (MS2) data only for some of the observed ions.

#### Internal standards

As part of the quality control across each experiment, three sets of internal standard mixtures which include compounds that cover a broad range of metabolic pathways such as amino acid metabolism, central carbon metabolism and nucleotide metabolism were run twice, before and after the cohort of samples was run. Detailed information about the internal standards used during the LC-MS experiments is included in the Supporting Information (S1 Table).

### Study workflow

**Fig 1:**
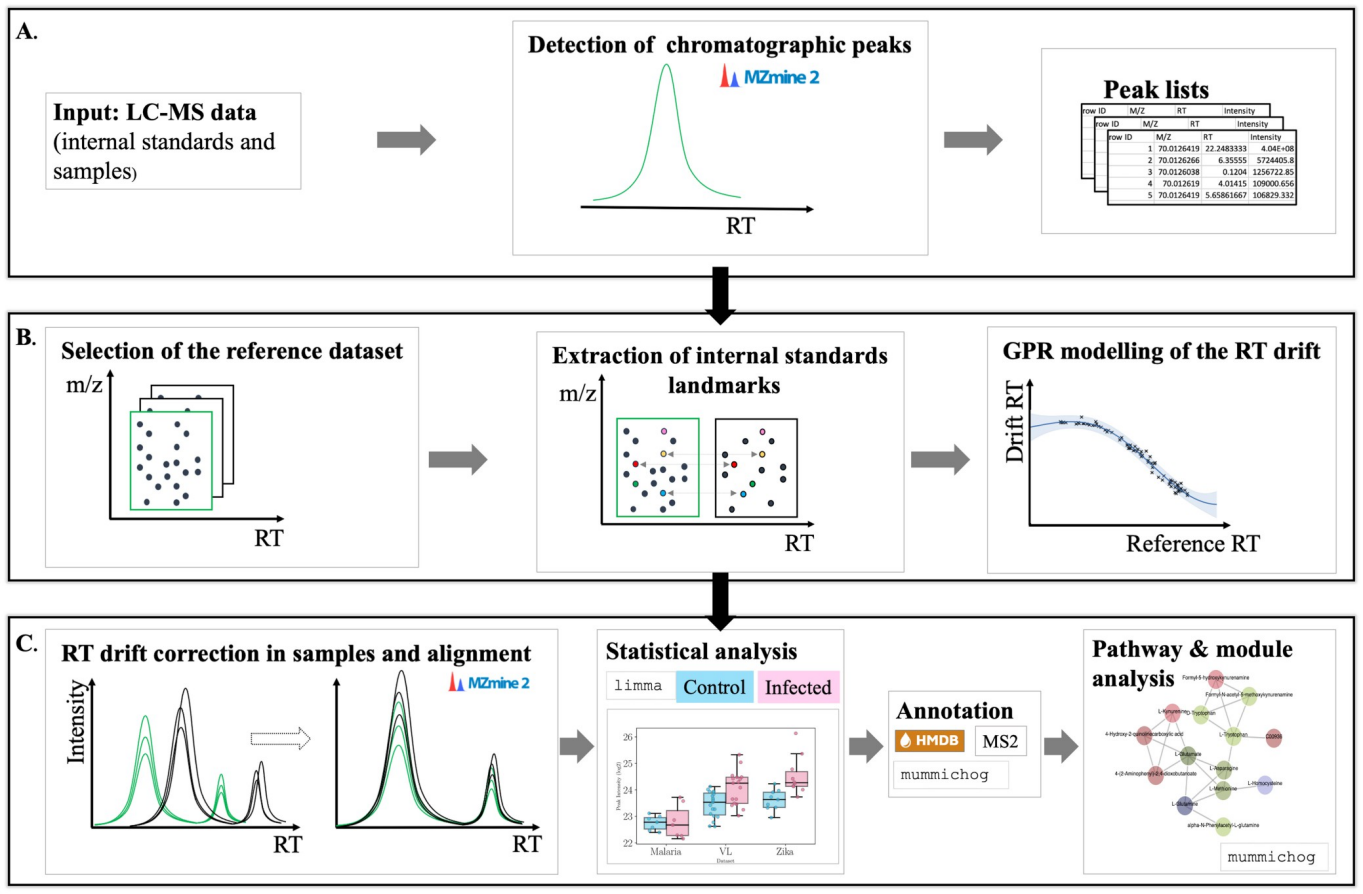
Diagram representing the study workflow. **A.** Peaks were detected from input LC-MS data (mzML format), including the internal standards and serum samples, using MZmine2. Peakset lists containing ion information (m/z, RT, intensity) were obtained. **B.** Internal standards analysis. A reference dataset was selected and the RT drift in the other datasets was calculated and modelled using GP regression. **C.** Sample analysis. Based on the GPR models obtained for each dataset, the RT was corrected in each peakset list and alignment was done using MZmine2. Afterwards, statistical analysis focused on the intensity differences between the control and infected samples was performed using limma R package. This was followed by annotation and pathway analysis using mummichog.

The data analysis process is outlined in Fig 1.

#### Peak detection

The spectral data was processed by detecting the chromatographic peaks from the internal standards and serum samples LC-MS data. Peak detection was performed using wavelet transform from MZmine2 v2.40.1 [17] in batch mode. For mass detection, the noise level threshold was set at 1.0E4, whilst for the ADAP chromatogram builder, the group intensity threshold was set at 1.1E4 and the minimum group size at 5. The m/z tolerance, which is dependent on the instrumentation used, was set at 3 ppm.

#### Internal standards analysis

First, the reference dataset, D_*ref*_ = D_*Z*_, was randomly selected from the two datasets with the shorter run length. Next, a profile was created for each dataset characterised by (m/z, RT) of the extracted internal standards compounds from the peak lists obtained after the peak detection process. The profiles of the non-reference datasets, D_*M*_ and D_*VL*_, were then mapped to the reference dataset profile and the RT drift between each of the non-reference datasets and the reference dataset was determined and modelled using GP regression.

#### GPR modelling of the RT drift

For each non-reference dataset, the RTs from the (m/z, RT) profile created were regressed against their respective RT drift from the reference dataset values. In order to obtain a closer fit to the data but still maintain the variability, internal standard metabolite outliers were removed based on their RT drift from the reference profile using a z-score cutoff value of 2. For implementing the Gaussian Process models, the GPy python package, version 1.9.9 was used [18]. Model hyperparameter optimisation was done using multiple restarts (n = 10) with the GPy optimiser to avoid local minima. To determine which covariance function aids in fitting the data best in each case, cross-validation was performed, by stratifying and splitting the internal standards data in half for training and testing the model. The prediction accuracy score, mean absolute error (MAE) and mean squared error (MSE) were calculated in each case.

#### GPR corrected data

The GPR corrected RT times were obtained by adding the GPR predicted variables (posterior mean) to the initial RT values of each peakset from the non-reference dataset.

#### Detection of alignment RT window parameter

The alignment of the peak lists was performed using the JoinAligner module from MZmine2. The optimal RTWindow parameter was determined by aligning the internal standards peak lists RTWindow values ranging from 0.01 min to 2 min. For each of the three internal standards sets the total number of peaks aligned and the total number of internal standards that align for each RTWindow value across the datasets was calculated. In order to determine the optimal RTWindow for all datasets, the value for which the alignment results in the lowest total number of peaks aligned and highest number of internal standards metabolites was selected for further analysis.

#### Sample analysis

The GPR models obtained in the previous stage were applied to each serum sample peak list by correcting the RT values for each peak as detailed above. The RT corrected peak lists were then aligned using the RTWindow value previously obtained. The final list of aligned peaksets was first processed by filtering out the peaksets based on the percentage of missing values from each dataset. An arbitrary cut-off value of 50% was used. Data imputation using the K-nearest neighbours method (k = 3) was performed solely for visualisation purposes [19].

#### Statistical analysis

The statistical analysis focused on the intensity differences between the sample peak lists belonging to the control and infected groups from all three datasets. The intensities were log2 normalised and modelled using linear regression from the limma R package, where blocking was used to adjust for the intensity variability between the different datasets. The output of this analysis was a list containing all the peaksets and their respective p-value, Benjamini-Hochberg (BH) adjusted p-value and logarithmic fold change (logFC) between the two conditions.

#### Feature annotation and pathway analysis

The MS2 spectra from each dataset was aligned to the final filtered peaksets list. A profile characterized by (m/z, RT, ms2spec) was created for the possible adducts/fragments for each peakset (S2 Table) and several methods were used for feature annotation. First, annotation was performed using the internal standards information by mapping the peakset profile against the internal standards (m/z, RT) profiles. Next, they were mapped against metabolite information extracted from the Human Metabolome Database (HMDB). Where one or more spectra was aligned to a peak, the attached spectrum/spectra was compared against experimental LC-MS spectra from HMDB and the match with the highest cosine similarity score was used to annotate the peak. For pathway and activity network analysis mummichog version 2.3.3. was used [20].

#### Code

All of the analysis was performed in python programming language (https://github.com/anamaria-uofg/mma).

### Methodology evaluation

The peakset lists with no GPR correction were aligned and the same workflow was applied to their analysis. The results obtained were compared with the results of the GPR modified data. Also, the datasets were individually analysed and their filtered peakset lists were then intersected to determine whether any commonality can be found in this way. Additionally, we evaluated the alignment process using the available MS2 data. If two compounds with similar m/z and RT break down into the same fragments (during LC-MS analysis), then it is highly likely they represent the same compound. Therefore, if a peakset had multiple highly similar MS2 spectra from different datasets, it is likely that the peaks were aligned correctly. In order to measure the similarity between two MS2 spectra, cosine similarity score implemented in mass-spec-utils was used [21]. In order to evaluate the alignment process using MS2 data, the spectral similarity between the MS2 profiles was computed when more than one spectrum was aligned to one peakset. In order to establish a ground truth, for each of the actual spectral similarity score (good spectral similarity score), the mean of the corresponding distribution of random spectral similarity scores (bad spectral similarity score) was calculated. These were obtained from spectra of peaksets with similar m/z (absolute tolerance = 0.01), but different RT (Δ RT = 40 s).

## Results

### Internal standards analysis

#### Correlation between the RT drift and other variables

Correlation between the RT drift and the characteristics of the dataset profiles was checked. For all datasets the highest correlation was found with the RT from each dataset profile. Based on this information, the training (70%) and test (30%) data were split and stratified in 4 equal length bins. Following cross-validation, the kernel with the highest accuracy and lowest MAE was chosen. The final model was fitted using the selected kernel and optimised using multi-start in order to deal with possible bad local minimum.

#### GPR modelling of the RT drift

**Fig 2:**
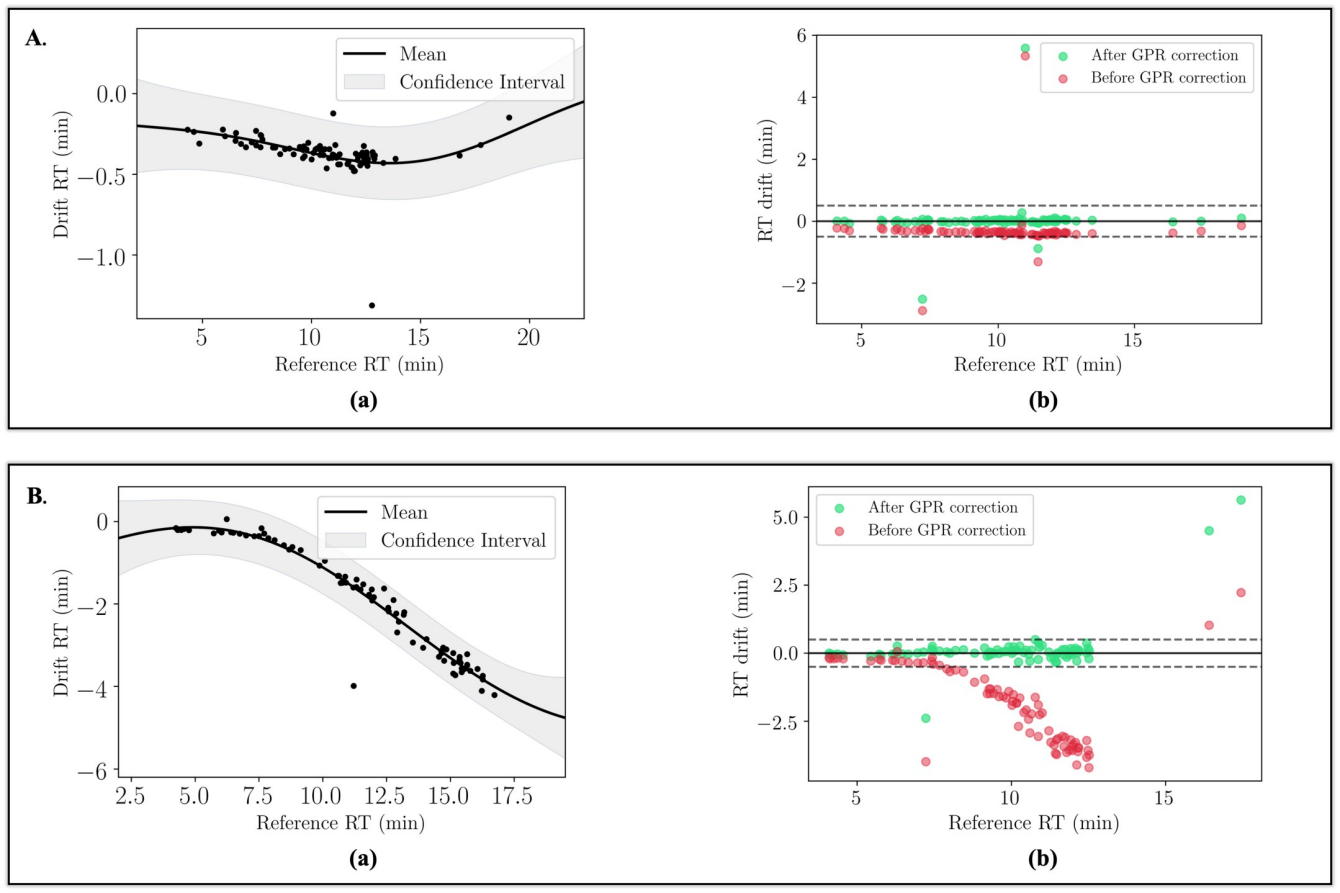
Modelling the RT drift in the two datasets D_*M*_ and D_*VL*_. **A.** Modelling the drift in D_*M*_ using an RBF kernel. Graph (a): Mean and 95% posterior confidence with optimised hyperparameters: RBF variance = 0.07, RBF lengthscale = 6.84. Graph (b) illustrates the RT drift in D_*M*_ before and after correction of the retention times using the GPR model. **B.** Modelling the drift in D_*VL*_ using a composite RBF + MLP kernel. Graph (a): Mean and 95% posterior confidence with optimised hyperparameters: MLP variance = 3.99, MLP weight variance = 2.02e7, MLP bias variance = 5.56e-309, RBF variance = 7.44, RBF lengthscale = 9.41. Graph (b) illustrates the RT drift in D_*VL*_ before and after correction of the retention times using the GPR model.

Both simple kernels, such as RBF, multilayer perceptron (MLP) and cosine kernels, and composite kernels were tested to determine which ones best fit the data (Fig 2). Composite kernels refer to multiple simple kernels combined either by addition or multiplication.

For D_*M*_ the mean retention time drift in comparison to the reference dataset was 19.85 s and the highest RT drift between two peaks belonging to the same ion was 319.74 s. Following cross-validation an RBF kernel was selected as the best for fitting the RT drift in D_*M*_ with an accuracy of 0.99, MAE = 0.06 and MSE = 0.03. The two hyperparameters of the RBF function, i.e. variance and lengthscale, were optimised to 0.07 and 6.84 respectively (Gaussian noise variance = 0.01) (Fig 2A).

For D_*VL*_ the mean retention time drift was 112.03 s and the maximum RT drift was 252.54 s. For the drift in D_*VL*_ a composite kernel RBF+MLP was chosen for fitting the data with an accuracy score of 0.995, MAE = 0.14 and MSE = 0.03 (Fig 2B). The hyperparameters after 10 optimisation restarts were: MLP variance = 3.99, MLP weight variance = 2.02e7, MLP bias variance = 5.56e-309, RBF variance = 7.44, RBF lengthscale = 9.41 (Gaussian noise variance = 0.1).

#### RTWindow value choice for JoinAligner module

For D_*M*_, 94.8% of the maximum number of internal standards in common with the reference dataset are aligned at RTWindow = 0.25 min after RT drift correction, as opposed to 19.48% before drift correction. Whereas, for D_*VL*_ the maximum number of metabolites which are aligned after drift correction are obtained at RTWindow = 0.5 min for which 92.67% of the metabolites align, as opposed to 17.64% before correction. At a RTWindow value of 0.25 min 83.82% of the metabolites align (S1 Fig). Based on these results, the optimal RTWindow parameter for further alignment of the samples was selected at RTWindow = 0.5 min.

### Methodology evaluation

#### GPR corrected data vs original data

Results obtained for the +ve mode data are presented. In total, 625 peaksets remained after filtering the GPR corrected data, as opposed to 344 peaksets in the non-corrected data. Most of the peaks which are aligned only in the GPR modified data have the retention time in the range of (7,13) minutes.

#### Individual datasets alignment

For D_*Z*_, 2305 peaksets remain after filtering, out of which 3 are significant. For D_*VL*_ 2392 peaksets remain after filtering, out of which 775 are significant. For D_*Z*_ 2152 peaksets remain after filtering, out of which 5 are significantly different. When intersecting the significantly different peaksets, there is no peakset found to be in common between all three datasets. Therefore, aligning the peaksets together increases the number of samples, increasing statistical significance.

#### MS2 data

Due to the fragmentation strategy employed in each experiment, MS2 spectra were available only for a small percentage of the data. From the filtered peaksets there were 217 peaksets with MS2 spectra aligned, out of which 141 had an MS2 spectrum from one dataset attached to it, 65 peaks had MS2 spectra from 2 datasets attached and 11 peaks had MS2 spectra from all 3 datasets. The majority of peaksets were fragmented only in one dataset. The bad spectral similarity scores attached to peaksets with the same m/z mainly have a lower similarity score than the good scores (87%), which shows that the alignment worked optimally for the majority of peaksets (S3 Fig).

### Sample analysis

#### Sample alignment

For the sample alignment, the −ve ESI mode data was processed in the same way as the +ve ESI mode data which was presented above. The JoinAligner module was run with RTWindow = 0.5 min in order to align all 74 samples across the three datasets. Following alignment there were 37,220 peaksets in +ve ESI mode and 24,729 in −ve ESI mode. After filtering out the peaksets with more than 50% values missing in any one dataset, only 1.68% of the total number of peaksets, i.e. 625, remained. A similar percentage was obtained in the case of the negative mode data where 1.85% (459) of the total number of peaksets remained. The differential expression analysis resulted in 207 significantly different (BH adjusted p-value<0.05) features and 159 features in the positive and negative mode, respectively.

#### Overview of significantly modified compounds

**Fig 3:**
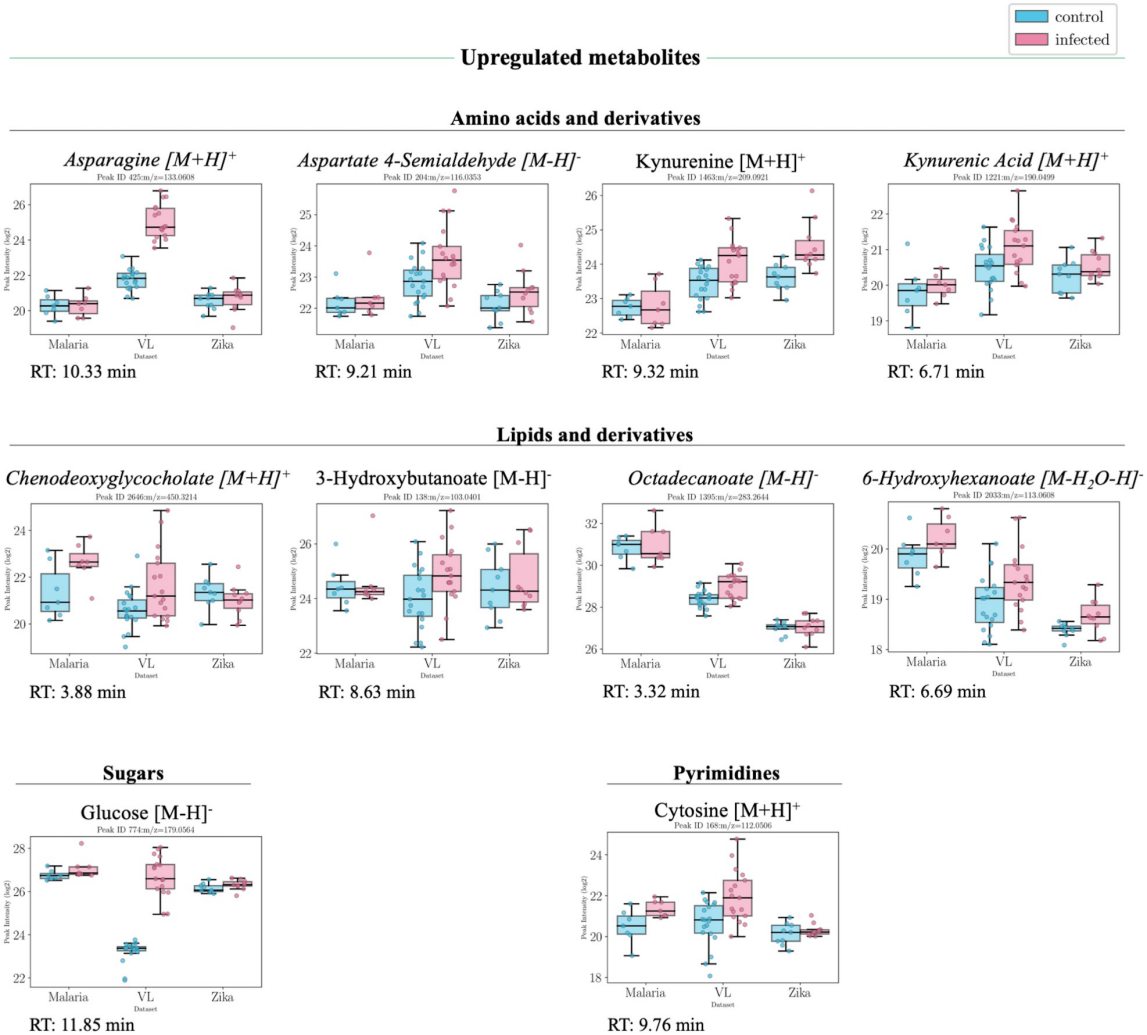
Boxplots of putatively annotated compounds which were upregulated in infected patients. Overview of putatively annotated metabolites which are statistically significant (p-val<0.05) and present a general upward trend in all three datasets, i.e higher intensities in infected patients. The metabolites are grouped based on their class or subclass according to HMDB. In this case, metabolites from amino acids and derivatives, lipids and derivatives, sugars and pyrimidines were identified. Values from both positive and negative ESI modes are presented from left to right in ascending order of their p-value. Metabolites in italic font are only annotated using mummichog.

**Fig 4:**
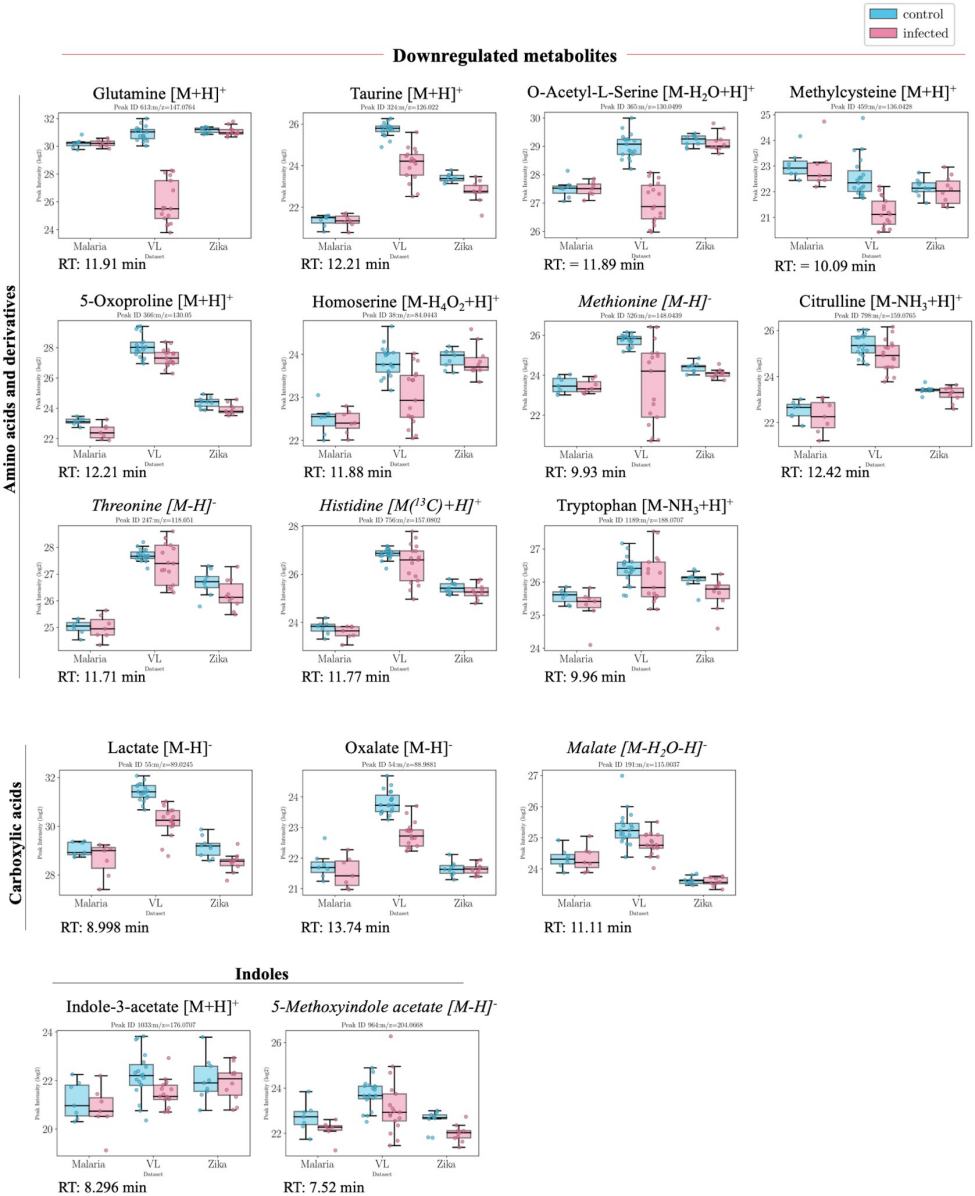
Boxplots of putatively annotated compounds which were downregulated in infected patients. Overview of putatively annotated metabolites which are statistically significant (p-val<0.05) and present a general downward trend in all three datasets, i.e. lower intensities in infected patients (with p-value<0.05). The metabolites are grouped based on their class or subclass according to HMDB. In this case, metabolites from amino acids and derivatives, carboxylic acids and indoles were identified. Values from both positive and negative ESI modes are presented from left to right in descending order of their p-value in each group. Metabolites in italic font are only annotated using mummichog.

**Fig 5:**
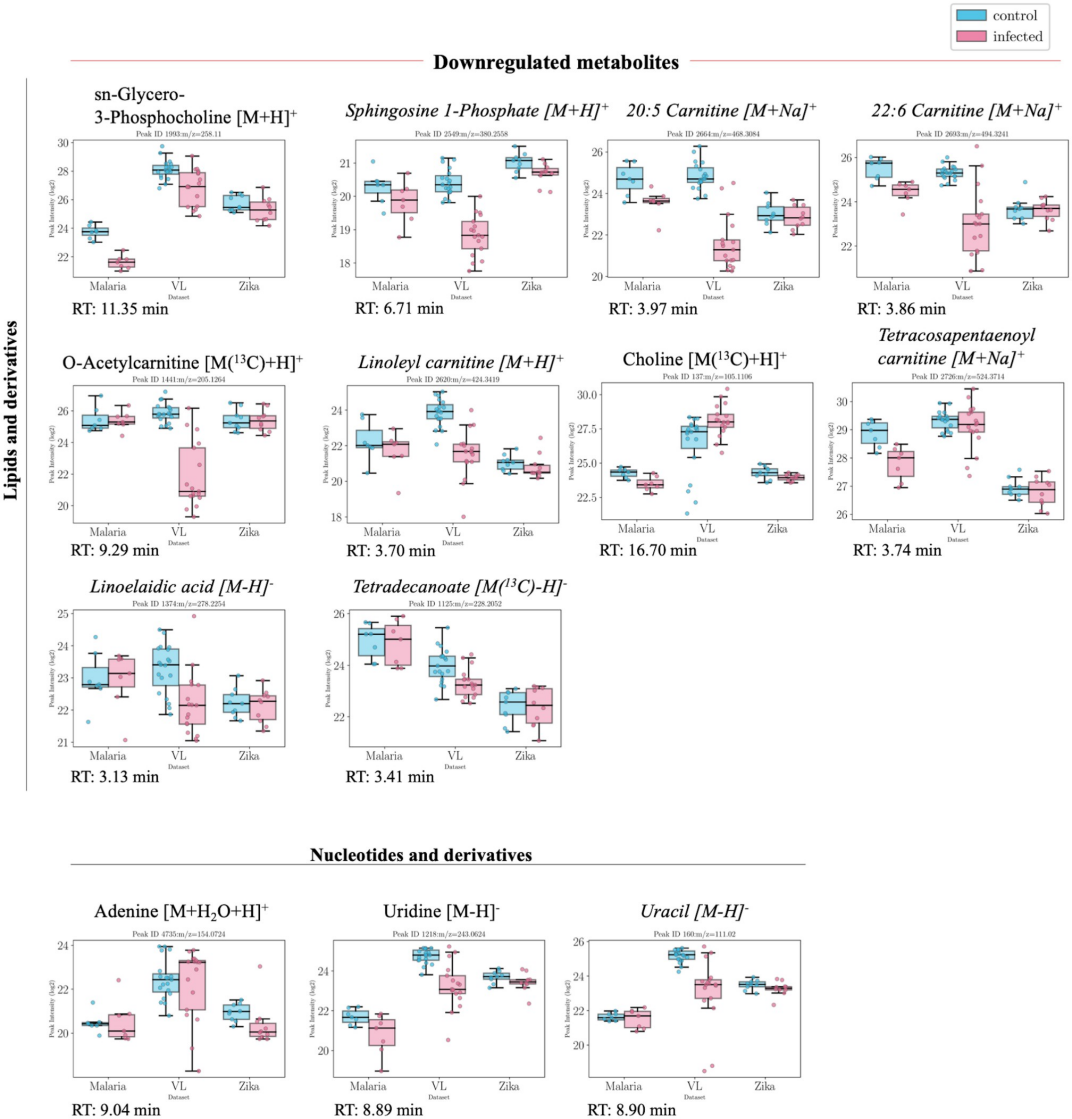
Boxplots of putatively annotated compounds which were downregulated in infected patients (continuation). Overview of putatively annotated metabolites which are statistically significant (p-val<0.05) and present a general downward trend in all three datasets, i.e. lower intensities in infected patients (with p-value<0.05). The metabolites are grouped based on their class or subclass according to HMDB. In this case, metabolites from lipids and derivatives and nucleotides and derivatives were identified. Values from both positive and negative ESI modes are presented from left to right in descending order of their p-value in each group. Metabolites in italic font are only annotated using mummichog.

The significant features with putative annotations either by standard matching, MS2 profile matching or mummichog analysis, that present common trends based on the logFC values between the two conditions (disease vs control) from each individual dataset, are presented in Figs 3, 4, 5 accompanied by the information in S3 Table. Based on this, a general trend over the three datasets was established. For the +ve ESI mode data, 30 peaksets presented a general upward trend (all datasets have logFC>0) out of which 11 were statistically significant and 150 peaksets presented a general downward trend out of which 69 were significant. For the −ve ESI mode data, 46 peaksets presented a general upward trend out of which 25 were statistically significant and 115 peaksets presented a general downward trend out of which 74 were significant.

A table containing the results following the statistical analysis with annotation is included in the Supporting Information (S2 Table). A large proportion of the features elute in clusters of similar retention time, indicating that they might be related, e.g. fragments or adducts of the parent ion. The list of ions used to calculate the possible adducts/fragments of each peak are included in the Supporting Information (S4 Table). Common adducts include gain of H_2_O, Na or K, isotopologues (C^13^, S^34^) and common fragmentation patterns include loss of CO, H_2_O, HCOOH, NH_3_, C_3_H_4_O_2_, H_4_O_2_.

#### Pathway analysis results

Following mummichog pathway analysis for both the positive and negative ionisation modes 20 KEGG pathways were found to be statistically significant. The pathway analysis revealed a significant impact of the studied infectious diseases on nitrogen metabolism with tryptophan metabolism predominant (Table 2). Following the modular analysis, the activity network for the +ve ESI mode is also centered around tryptophan metabolism, specifically the kynurenine pathway, which is discussed in more detail in the next section (S4 Fig). For the −ve ESI mode data the activity network also involves tryptophan metabolites and, additionally, citric acid cycle metabolites.

**Table 2:** Significantly altered metabolic pathways (p-val<0.05) following mummichog analysis of the negative and positive ionisation mode data.

KEGG Pathway	p-value	ESI mode
Nitrogen metabolism	0.000672	+
Tryptophan metabolism	0.00084	+
Alanine and Aspartate Metabolism	0.002017	+
Pyruvate Metabolism	0.00521	-
Vitamin B3 (nicotinate and nicotinamide) metabolism	0.005546	+
Pyrimidine metabolism	0.007226	+
Glycolysis and Gluconeogenesis	0.008151	-
Carnitine shuttle	0.009243	+
Glycerophospholipid metabolism	0.013024	-
Glycosphingolipid metabolism	0.013024	-
Methionine and cysteine metabolism	0.015965	+
Aminosugars metabolism	0.022939	+
Purine metabolism	0.022939	+
Bile acid biosynthesis	0.031846	+
Fatty Acid Metabolism	0.031846	-
Putative anti-Inflammatory metabolites formation from EPA	0.031846	+
Androgen and estrogen biosynthesis and metabolism	0.03924	-
C21-steroid hormone biosynthesis and metabolism	0.03924	-
Vitamin B1 (thiamin) metabolism	0.03924	-
Glutathione Metabolism	0.04445	+

This is the merged output obtained following mummichog analysis of both +ve and −ve ESI mode data. The p-values are based on EASE score (a variant of Fisher exact test) and adjusted on the cumulative distribution function of the Gamma model [20].

#### Tryptophan metabolism

**Fig 6:**
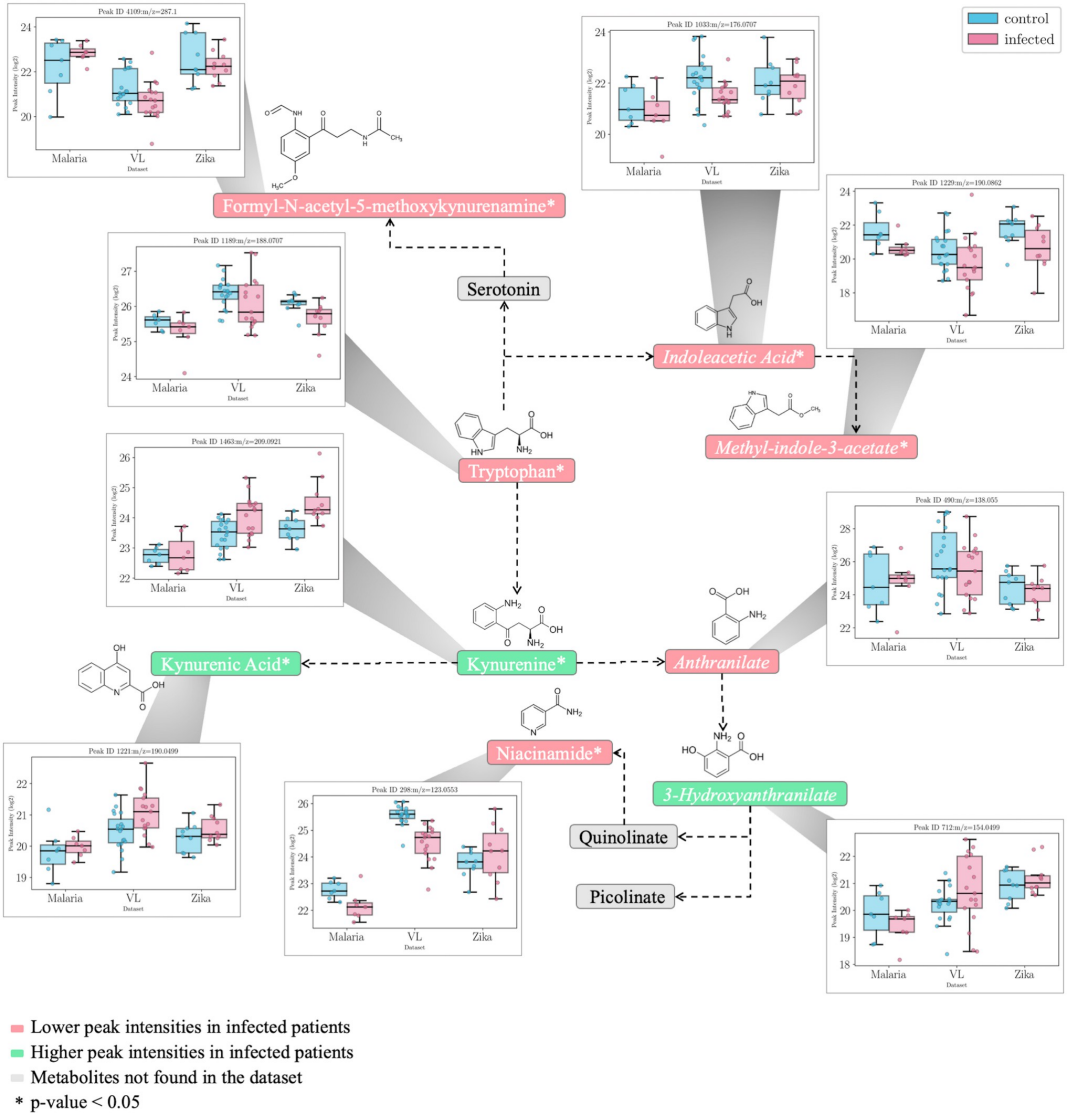
Tryptophan metabolism and the changing metabolites from each dataset. Intensity values are represented as lg2 values. The metabolites were mapped against the Kyoto Encyclopedia of Genes and Genomes (KEGG) pathway map hsa00380. Metabolites in italic font are annotated following mummichog analysis or the HMDB matching method, while the rest are annotated using the internal standard metabolites information. The boxplots represent the intensities of all the samples in each condition (red = infected, blue = control) in all three datasets.

Focused analysis on the tryptophan metabolic pathways represented in Fig 6 revealed significant decreases in all three datasets of tryptophan and tryptophan derivatives such as indoleacetic acid and methoxyindole acetate. Methyl indole acetate and formyl-N-acetyl-5-methoxy kyunernamine were also significantly decreased in the infected group with the exception of the Malaria dataset where the logFC value was slightly higher (Fig 6). In contrast, the kynurenine branch of tryptophan metabolism suggests an increased activation as kynurenine and kynurenic acid are present at higher levels in infected patients in all three datasets. 3-Hydroxyanthranilate was in general higher as well with the exception of the Malaria dataset were logFC value was slightly lower. Anthranilate levels were also reduced in infected patients across all datasets, although not reaching statistical significance. Nicotinic acid (−ve ESI mode) was also found in significantly lower levels in infected patients and nicotinamide was significantly lower, although the Zika dataset had positive logFC (Fig 4). It is important to note that unless otherwise stated, annotations are based on m/z alone.

Tryptophan metabolism has previously been associated with various agents of infection [22], particularly its flow through the kynurenine pathway which produces metabolites including kynurenate and nicotinamide adenine dinucleotide (NAD+). Of particular interest is the inverse relation between kynurenine and tryptophan, as the ratio between the two is used to measure the activity of the enzyme indoleamine-2,3-dioxygenase 1 (IDO-1) [23]. IDO-1 is the rate limiting step of the tryptophan pathway and it catalyses the breakdown of tryptophan to kynurenine. IDO-1 activity is also tightly regulated by interferon gamma (IFN-*γ*) activity [24]. Similarly, COX-2, an enzyme central to the fever process, can also be induced by IFN-*γ* [25]. The interplay between IDO-1 and COX-2 enzymes has been previously studied where inhibition of COX-2 enzyme has led to a downregulation in IDO-1 and decrease in kynurenine metabolites [26].

It could be hypothesised in this case, that the increased level in kynurenine and decreased levels of tryptophan indicate an increased IDO-1 activity, and subsequently an increased COX-2 activity. Due to the importance of these metabolites, further investigation was conducted in verifying the identity of the putatively annotated metabolites using the available MS2 spectral information.

#### Verifying the identity of tryptophan and kynurenine peaks using MS2 spectral information

**Fig 7:**
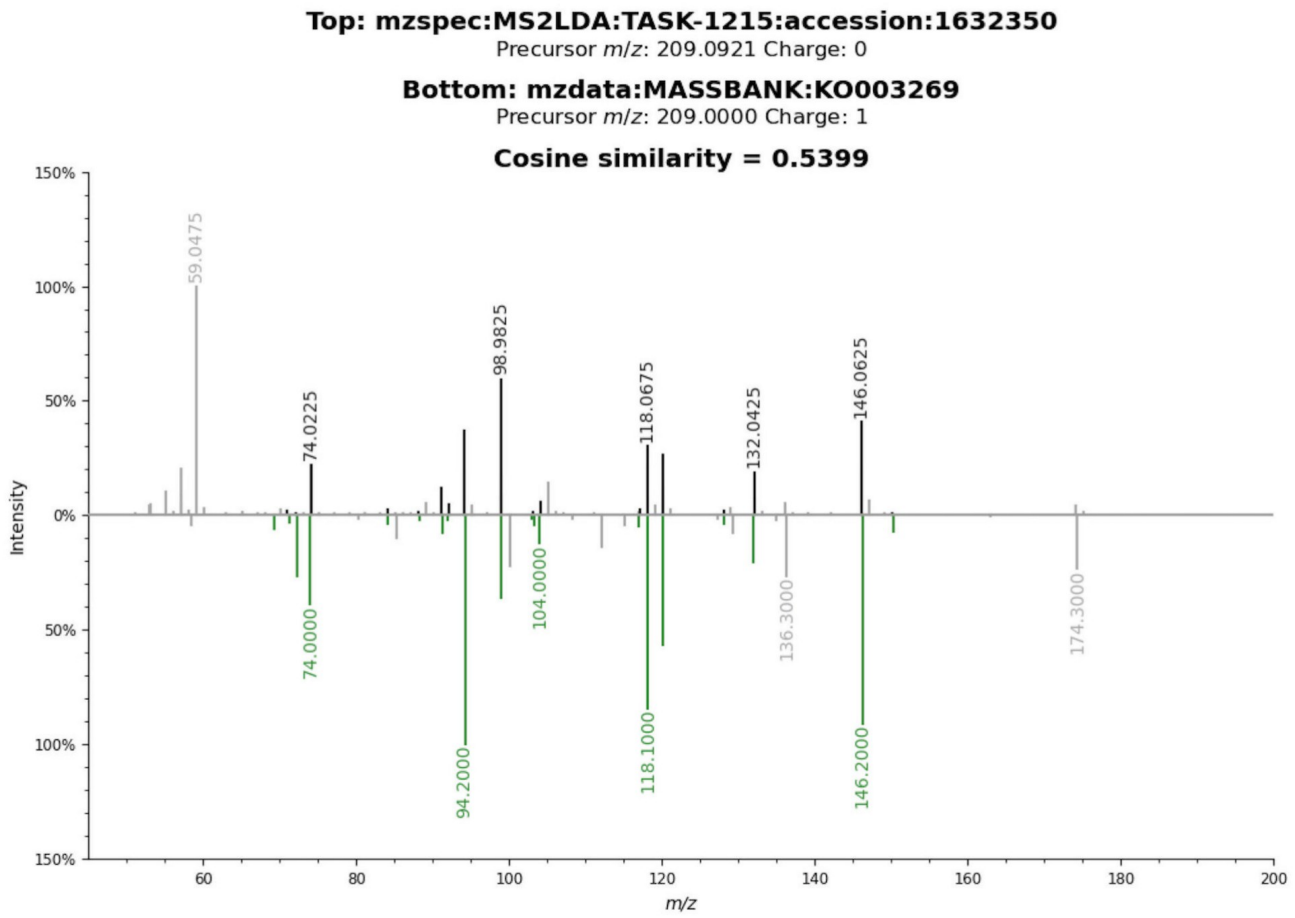
Peakset annotated as Kynurenine (M+H[1+]). using internal standards matching method, mummichog and HMDB matching method. The spectrum belongs to D_*VL*_ (resolver obtained from ms2lda.org) and it was matched against experimental LC-MS MS2 information from MassBank compound KO003269 with a cosine similarity score of 0.54 (fragment tolerance = 0.2) (Metabolomics spectrum resolver plot).

**Fig 8:**
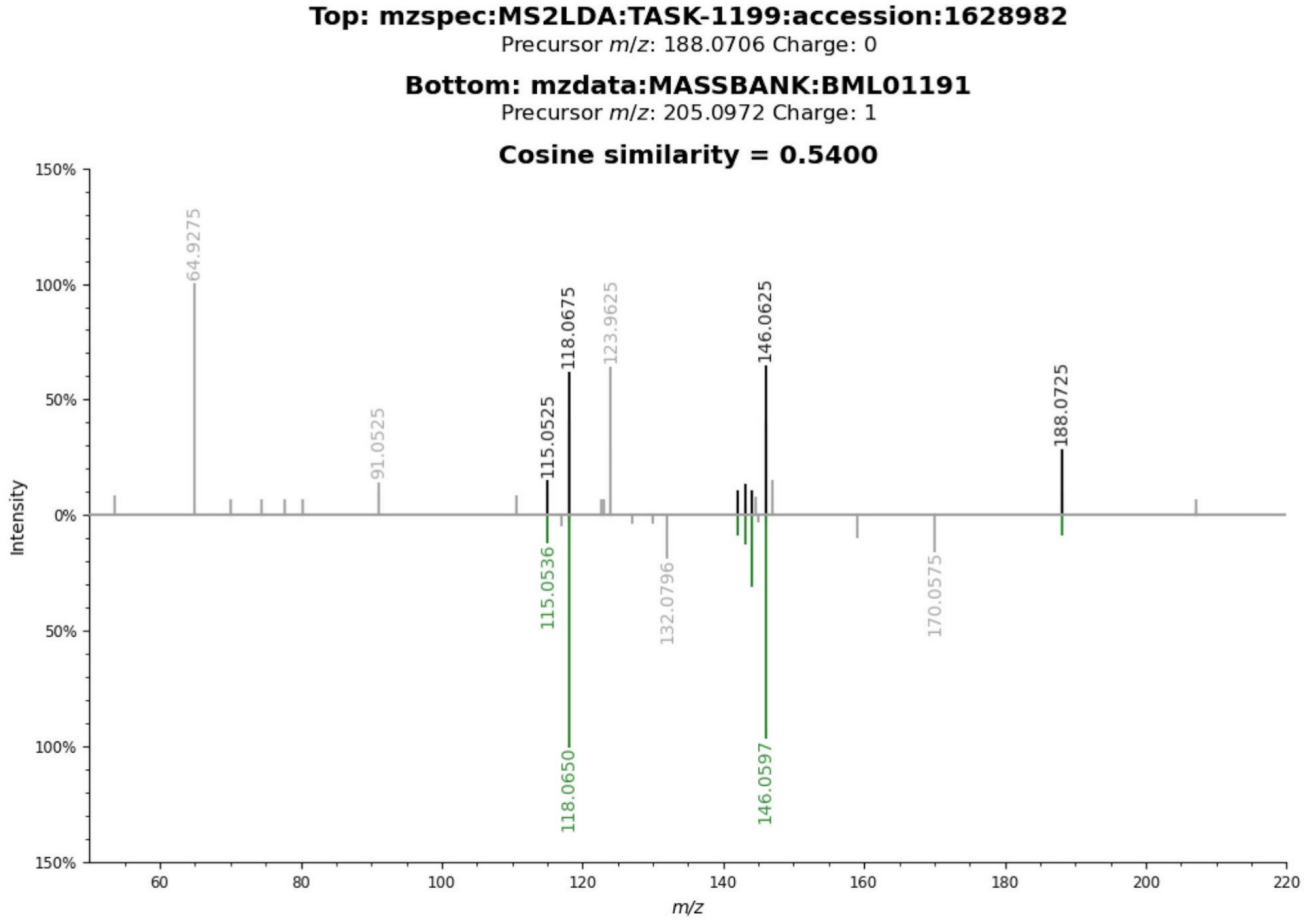
Peak annotated as L-Tryptophan fragment with loss of ammonia (M-NH3+H[1+]). using internal standards matching method, mummichog and HMDB matching method. The loss of ammonia from protonated tryptophan was observed as the primary fragmentation pathway in gas-phase reactions [28]. The spectrum belongs to D_*Z*_ and it was matched against experimental LC-MS MS2 information from MassBank BML01191 compound with a cosine similarity score of 0.55 (fragment tolerance = 0.2) (Metabolomics spectrum resolver plot).

As both the tryptophan and kynurenine peaks had spectral information from one out of the three datasets, this was used to verify the identity using the cosine similarity score employed in Metabolomics spectrum resolver [27]. Similarity scores of 0.54 and 0.55, respectively, were obtained for kynurenine and tryptophan fragment (with loss of ammonia) (Figs 7 and 8).

### Other affected metabolic pathways

#### Amino acid metabolism

Other significantly affected pathways relate mostly to other amino acids including those from alanine, aspartate and glutamate metabolism, methionine and cysteine metabolism and glutathione metabolism (Table 2). Fig 6 indicates a clear reduction in a number of amino acids and their metabolites particularly glutamine and related metabolites. In immune cells, glutamine is converted through glutaminolysis to glutamate, aspartate and alanine by undergoing partial oxidation [29]. This could explain the decrease in glutamine and increase in aspartate in the infected group in all three datasets. Similarly, another glutamine derived metabolite, asparagine, was found to be increased in infected patients in all three datasets. Glutamine also acts as a precursor for citrulline which plays an important role in arginine biosynthesis in the urea cycle. Citrulline levels were significantly lower in the infected group from the three datasets.

Altered glutathione metabolism accompanied by a dysregulated methionine and cysteine metabolism was also observed. The plasma levels of the amino acid 5-oxoproline (pyroglutamic acid) were also lower in the infected patients in comparison to healthy controls. In addition to decreased methionine, methylcysteine was also decreased in the infected patients. A precursor of cysteine, o-acetylserine was also decreased in the infected group alongside threonine and homoserine. Taurine, another metabolite derived from cysteine metabolism, was also found to have significantly lower levels in infected patients in the metadataset. The decrease in reduced thiols, may arise due to increased levels of oxidative stress in response to infection. There has been previous evidence which suggests that disruptions of the redox homeostasis affect normal body temperature [30].

Other amino acids presenting lower levels in all three datasets in the infected group but not with statistical significance include: beta-alanine, proline and betaine. In contrast, several amino acids and their relatives presented overall higher levels in infected patients including carnitine, tyrosine and leucine.

#### Central carbon metabolism

A significant increase in glucose was also noted across datasets (Fig 3) indicating alterations in central carbon metabolism. Indeed, perturbations to the glycolytic process and citric acid cycle, in particular, were confirmed in −ve ESI mode data particularly with significant decreases in lactate and malate. The significant increase in glucose is related to stress-induced hyperglycaemia which occurs in cases such as fever and infectious diseases [31]. This occurs due to the interaction between proinflammatory cytokines (TNF-*α*, IL-1, IL-6), the hypothalamic-pituitary axis and the noradrenergic system [31].

#### Lipid metabolism

Lipid abnormalities were also noted in the sera of infected patients, which demonstrated significant changes in the fatty acid metabolism, where there was a significant decrease mainly in particular acylcarnitines (Fig 3). Fatty acids, 3-hydroxybutanoate, 6-hydroxyhexanoate and octadecanoate were, on the other hand, all increased. This could be related to the previously mentioned increase in carnitine and subsequent decrease in acetylcarnitine levels which might indicate an increased activity in releasing fatty acids. Short-chain fatty acids are known to have a protective role and play a beneficial part in reducing endothelial activation, which leads to a reduction in proinflammatory cytokine production and adhesion molecule expression [32].

Sphingolipid and glycerophospholipid metabolism were also affected with sphingosine 1-phosphate being lower in all datasets. This might be indicative of liver damage, which is also reflected in the significantly modified levels of bile acids and taurine [33]. Bile acid biosynthesis metabolism also seems to be affected with taurine levels decreasing significantly in infected patients and chenodeoxycholate being significantly increased in infected patients. Choline and its derivatives were also downregulated in infected patients, which might also be related to macrophage metabolism [34].

Sphingolipids are not only important cellular membrane components, but they also have a dynamic role in cellular signalling and are involved in processes such as proliferation, endocytosis, necrosis, apoptosis and cellular migration [35]. Key molecules in sphingolipid signalling are: ceramide, sphingosine and sphingosine-1-phosphate (S1P). S1P is produced during inflammation or following tissue damage and it has been reported that pathogens affect S1P signalling via sphingosine kinase/S1P axis [36]. S1P exists both in the intracellular and extracellular pool, particularly in plasma. Plasma S1P has been shown to regulate various processes related to pathogenesis [37]. According to [37] reduced plasma S1P levels were associated with malaria severity in mice. There is also evidence that S1P bioavailability could have a role in attenuating endothelial damage. S1P also known to induce NO release from endothelial cells [38].

Linoelaidic acid was also putatively annotated. This is an isomer of linoleic acid, so without further confirmation the feature could also be linoleic acid, a precursor to eicosanoids. It was found to be significantly lower with a general downward trend in all datasets. Another feature was annotated as linoleic acid which was annotated both by *mummichog* and MS2 spectral validation and was also decreased in infected patients, albeit without reaching statistical significance, apart from D_*Z*_ where logFC_*i*_ was positive (0.4). Linoleic acid is a precursor for arachidonic acid from which prostaglandins and other bioactive eicosanoids are synthesised. In the context of fever, it could be hypothesized that an increased production of PGE2 due to the activation of COX-2 causes an increased turnover of arachidonic acid with a subsequent increased usage of linoleic acid, hence the decreased levels in serum. The LC-MS platform used here does not readily detect arachidonic acid or its products.

#### Nucleotide metabolism

Pyrimidine metabolism is significantly affected with cytosine being significantly higher in infected patients. Viral infections are known to cause significant metabolic changes in host cells, such as upregulation of pyrimidine nucleotide biosynthesis [39]. Uracil and its derivative, uridine, on the other hand, were found to be significantly decreased in all three datasets in the infected group. Purine metabolism is also affected, with adenine being significantly lower in infected patients.

## Discussion

### Alignment of disparate LC-MS datasets

In this study we have identified a set of metabolic compounds, some of which were putatively annotated, which acted in a similar manner in three different LC-MS case-control datasets including infectious diseases associated with fever. The diseases were malaria, Zika virus disease and visceral leishmaniasis. The algorithm behind the integration of the three datasets was based on alignment using the raw files with corrected RT. Correspondence algorithms have been studied and variation in retention time can be classified into system variation and component level variation [9]. The system variation is general to the whole run, whereas component level variations are specific to a single analyte or a group of analytes, so they cannot be modeled using monotonic functions.

Recent studies which have explored the retention time drift problem in the context of large sample sizes have led to several algorithms being developed to correct the problem. One study [10] presented a method for aligning the samples at a population scale (N = 2895 human plasma samples) by correction of the non-linear retention time shift inside the raw files, in a manner similar to that developed here. In order to determine the retention shift between samples, isotope labelled standards were used to allow modelling of the shift to correct raw files for further peak detection and alignment. Our approach also corrects raw files prior to peak detection and alignment but does not require heavy isotope labelled standards. Another study in which alignment between samples from a large-scale dataset (N = 1000) is addressed [40] proposed a profile-based alignment algorithm which uses a graphical time warping method to correct the retention times for mis-aligned features.

Several have used endogenous reference peaks to model the retention time shift between sets of samples. Li et al. [41] for example, used adjacent tandem mass spectrometry information to select endogenous reference compounds to model RT drift. A recently published study [42] also used internal reference compounds selected based on their m/z and intensity to model the RT difference between two aligned LC-MS datasets using a generalised additive model.

The advantage of aligning multiple LC-MS datasets at a retention time level over comparing previously annotated results from different datasets is that both putatively annotated and unannotated compounds that act in the same way or uniquely to each disease can be detected. Additionally, as the overall sample size is increased, by including separate but related datasets, statistical robustness of the analysis is enhanced, provided assumptions on the underlying similarity in responses of disparate datasets (for example separate pathogen related infections here) are robust. A common limitation in many LC-MS biomarker discovery studies introduced by the small sample size is thus overcome. Annotation using MS2 information is also improved, as some datasets contain MS2 spectra for peaksets which are absent in other datasets. This could be advantageous for datasets which have limited fragmentation data available, as it improves the chance of a peakset having MS2 spectra aligned, and thus the possibility for better annotation.

A limitation of our study was that the algorithm was only tested with datasets run in the same laboratory, on the same LC-MS platform and at the moment it is not known whether this method could be applied to metabolomics datasets run on different platforms. Annotation is also a limitation, as it is for metabolomics studies in general [43, 44]. It is to be noted that for some of the metabolites, the difference between the control group and the infected group was larger in one of the datasets than in the other datasets and in some cases this could have contributed to the statistical significance of the difference in the metabolites abundance in control as opposed to infected. This could be either due to the disease itself or its severity. In this case it is to be noted that D_*M*_ was an intervention study and the infection was controlled and less severe which might explain relatively lower logFC in the dataset. However, for this study only the metabolites which presented fold changes going in the same direction for all datasets were presented and discussed unless specified otherwise. Using disparate diseases of matched severity might identify even more common features, but our study was constrained by the availability of particular datasets.

### Pathways affected in common by infection with separate fever-related pathogens

The algorithm was written with the intention of comparing datasets related to infection, hence we sought differences between infected patients and healthy controls from all three datasets using linear regression from limma and mummichog to determine the biological network of activity and significantly affected pathways common to the infected samples group. This study offers a route to identify commonality in the metabolic profile in infected patients affected by pathogens that cause fever. At the centre of this meta-dataset stands the relationship between kynurenine and tryptophan related metabolites.

The currently proposed molecular basis for fever involves the following. The innate immune system is activated through pathogen recognition by toll-like receptors e.g. TLR-4. This initiates the production of pyrogenic cytokines (IL-6, IL-1*β*, TNF-*α*). These pyrogenic cytokines then act on the organum vasculosus of the laminae terminalis in the hypothalamus. At the same time, PGE2 is released from hepatic Kupfer cells via the activation of cyclooxygenase-2 (the rate limiting enzyme in the synthesis of prostaglandins). PGE2 also acts on the pre-optic nucleus in the hypothalamus leading to an elevated temperature set-point. Additional negative feedback systems prevent excessive elevation of body temperature via antipyretic cytokines (IL-1RA, IL-10, TNF-*α* binding protein) [1].

Based on the results obtained here, a possible connection between fever and the kynurenine pathway could be explained by the interplay between IDO-1 and COX-2. An activation of IDO-1 could lead to a decreased inhibition of COX-2 which in turn could lead to an increased activation of PGE2 release. The link between the two enzymes and inflammation has been previously studied [25]. Suppression of the COX-2 enzyme activity could also be decreased by the lower levels of nicotinamide in the infected group, as nicotinamide has been shown to influence the activity of COX-2 [45]. It is worth noting that serum metabolomics, as used here, detects only a faint echo of the changes that are occuring in specific cell types orchestrating inflammation in local anatomical sites associated with infection. Although PGE2 was not annotated in these datasets (PGE being difficult to detect using the platform used), linoelaidic acid and linoleic acid were putatively annotated and found to be decreased. Linoleic acid is a precursor of arachidonic acid and bioactive eicosanoids including PGE2. Decreased levels of linoleic acid in the infected group could corelate to its increased use in arachidonic acid and, thus, PGE2 production. Recent metabolomics investigations on SARS-CoV2 infection in coronavirus patients also pinpointed significant alterations in the tryptophan-kynurenine pathway [46, 47], with kynurenine levels increasing with disease severity.

Many of the other metabolites found here to have significant abundance level differences between the two groups have also been noted previously for roles in immunometabolism. Glutamine, for instance, plays a major role in the immune system, as a key energy source for immune cells and it is used at a higher rate during catabolic conditions such as sepsis or other infections [29]. Depletion of glutamine and also citrulline identified in this study could also be used as indicators of disease severity as suggested in [48]. Decreased oxoproline levels were also previously associated with a non-infectious fever-associated disease, Rheumatoid Arthritis [49]. Taurine is another amino acid which has previously been associated with inflammation and fever associated with diminished levels of the inflammatory cytokines TNF-*α* and IL-6 [50], and also related to lower body temperature when administered intracerebroventricularly [51].

## Conclusion

In conclusion, three LC-MS datasets obtained separately to investigate metabolic changes in Zika, malaria and VL infected patients were successfully aligned using fitted GP models for correcting the RT drift between them, determined by the RTs of the internal standards metabolites within each dataset. Following annotation and statistical annotation, compounds changing in abundance in similar ways were found across the different infectious diseases. Common dysregulation patterns were observed in metabolic pathways related to amino acid and carboxylic acids metabolism, lipid and nucleotide metabolism with kynurenine pathway from tryptophan metabolism being identified as the most significantly changed pathway.

## Supporting information

S1 File Details regarding the sample collection for each dataset. (PDF)

S1 Table Internal standards metabolites information. (XLSX)

S2 Table List of filtered peaksets. (XLSX)

S3 Table List of annotated peaksets represented in the boxplots. (XLSX)

S4 Table Table with positive and negative ESI mode ion adducts. (XLSX)

S1 Fig Checking the internal standards alignment. (TIF)

S2 Fig Chromatogram of corrected RT samples. (TIFF)

S3 Fig Methodology evaluation using MS2 data. (TIFF)

S4 Fig Mummichog activity networks. (TIFF)

S5 Fig Linoleic acid in control vs infected samples. (TIFF)
