# Supplementary figures and images for: Alignment of multiple metabolomics LC-MS datasets from disparate diseases to reveal fever-associated metabolites
Source: PLoS Negl Trop Dis. 2023 Jul 24;17(7):e0011133. doi: 10.1371/journal.pntd.0011133 (PMC10399774; doi:10.1371/journal.pntd.0011133)

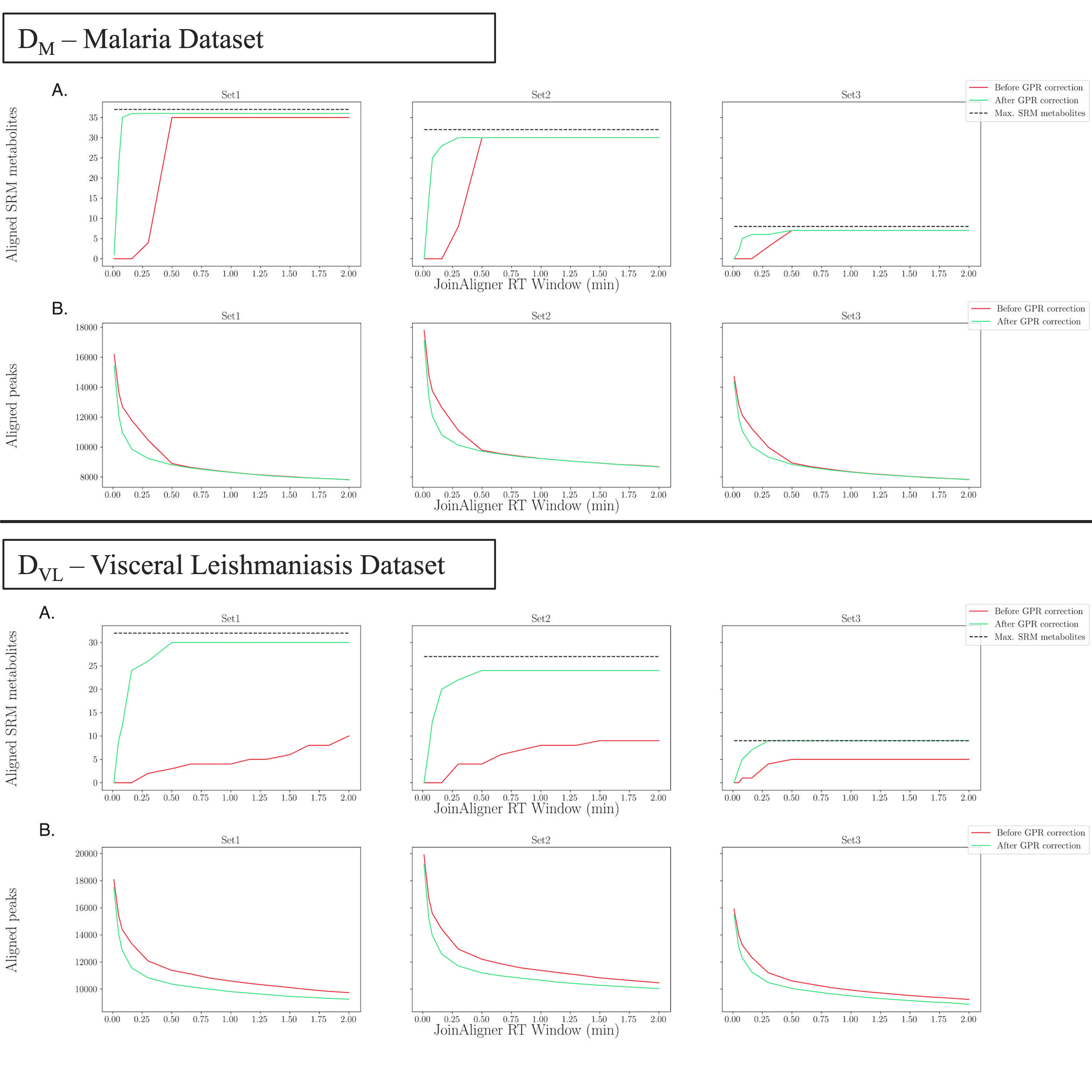

Supplement: S1 Fig — (TIF) [file pntd.0011133.s006.tif]

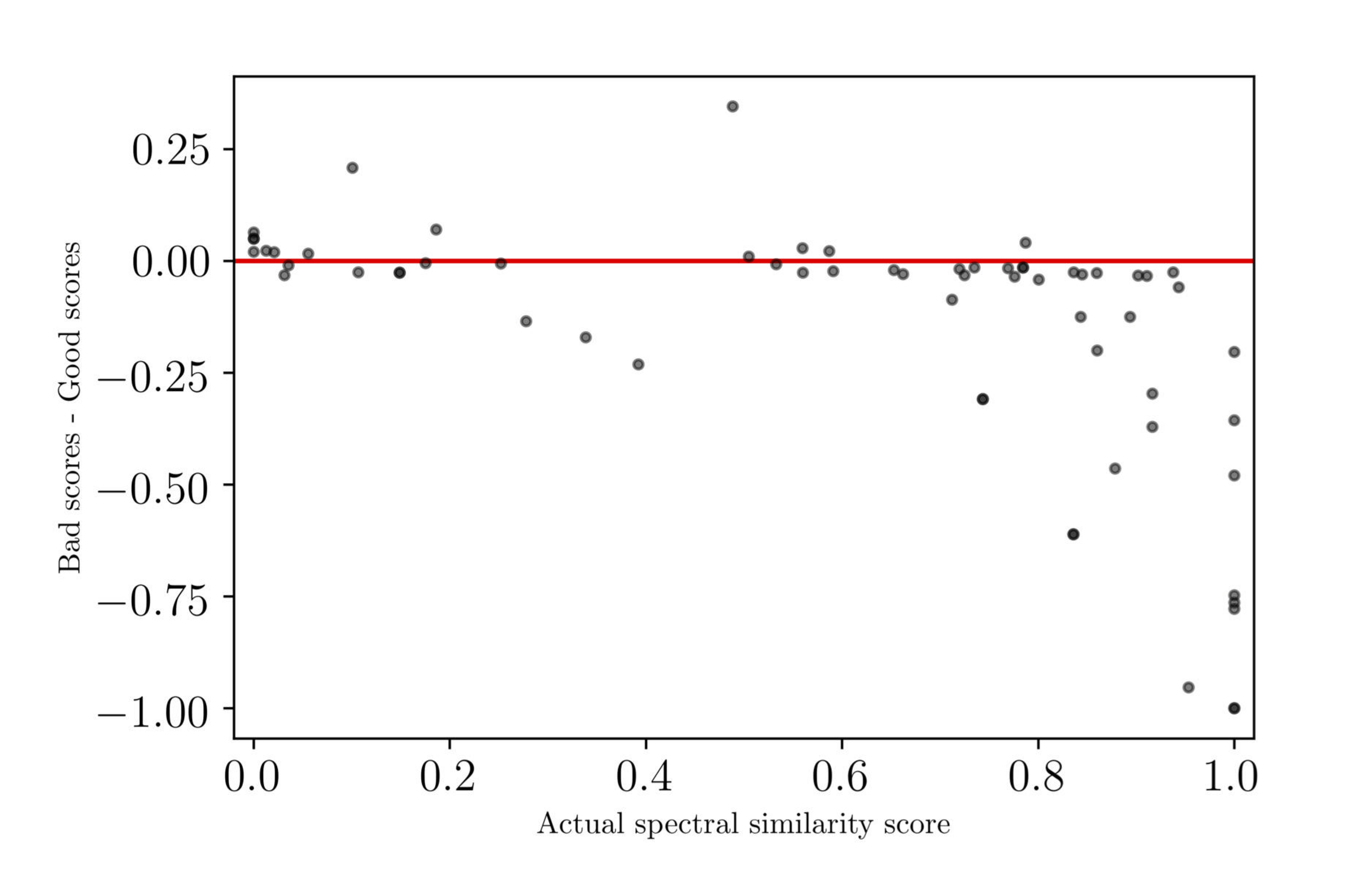

Supplement: S3 Fig — (TIFF) [file pntd.0011133.s008.tiff]
